# The accuracy of fixed intensity anchors to estimate lactate thresholds in recreational runners

**DOI:** 10.1007/s00421-025-05748-8

**Published:** 2025-03-15

**Authors:** Olli-Pekka Nuuttila, Piia Kaikkonen, Harri Sievänen, Tommi Vasankari, Heikki Kyröläinen

**Affiliations:** 1 The UKK Institute for Health Promotion Research, Kaupinpuistonkatu 1, 33500 Tampere, Finland; 2 Faculty of Sport and Health Sciences, University of Jyväskylä, Jyväskylä, Finland; 3 Tampere Research Center of Sports Medicine, UKK Institute, Kaupinpuistonkatu 1, 33500 Tampere, Finland; 4 Faculty of Medicine and Health Technology, Tampere University, Tampere, Finland

**Keywords:** Lactate threshold, Metabolic threshold, Endurance training, Exercise prescription

## Abstract

**Background:**

Endurance exercise intensities can be categorized into moderate, heavy, and severe domains based on physiological responses during incremental exercise testing. Since the evaluation of metabolic thresholds is not always possible, this study assessed the accuracy of fixed intensity anchors to estimate lactate thresholds.

**Methods:**

165 (64 females, 101 males) recreational runners performed a maximal incremental treadmill test. The first (LT1) and second (LT2) lactate thresholds were determined as percentages of maximum heart rate (HR), oxygen consumption (VO_2_), and running speed, alongside the rating of perceived exertion (RPE). Sex-specific mean values were used to determine the threshold intensities and to analyze the error magnitude for each method.

**Results:**

Females had a higher relative HR, VO_2_, and speed at LT1 compared to males (*p* < 0.001). In the pooled data, the mean absolute error for estimating LT1 varied from 0.6 to 0.8 km/h for speed and 4.9–7.4 bpm for HR, while LT2 errors ranged from 0.4 to 0.8 km/h and 2.8–5.2 bpm, respectively. The speed-derived estimations yielded the smallest error magnitudes, while the RPE-derived estimations were the least accurate. Estimating the maximum speed increased the respective errors to 1.0 km/h and 8.4 bpm for LT1, and to 1.1 km/h and 6.7 bpm for LT2.

**Conclusion:**

LT1 occurred at higher relative intensity in females, suggesting a need for sex-specific intensity anchors. The speed-derived estimates were the most accurate, but the estimation of maximum values impaired the prediction accuracy. Thus, the optimal method also depends on the availability of the maximum values.

**Supplementary Information:**

The online version contains supplementary material available at 10.1007/s00421-025-05748-8.

## Introduction

The intensity of physical exercise is a critical component of exercise prescription, affecting both the acute physiological responses (Nuuttila et al. 2021; Meyler et al. 2023) and chronic training adaptations (Helgerud et al. 2007; Inglis et al. 2024). While quantifying the amount of training is rather straightforward, the same does not always apply to exercise intensity. There are numerous ways to classify exercise intensity domains, the optimal choice depending also on the population and the objective of the assessment (Coates et al. 2023). In the context of endurance training, the most justified method is to divide the intensity domains based on metabolic thresholds (e.g., lactate and/or ventilatory) into moderate, heavy, and severe (Jamnick et al. 2020), also known as low/zone1, moderate/zone2, and high/zone3 (Seiler 2010). The moderate intensity domain is typically below the first metabolic threshold (1st lactate threshold = LT1; 1st ventilatory threshold = VT1), the heavy intensity domain between the first and second (2nd lactate threshold = LT2; 2nd ventilatory threshold = VT2) metabolic thresholds, while the severe intensity domain is between the second metabolic threshold and the intensity of the maximum oxygen uptake (VO_2max_) (Jamnick et al. 2020). In addition to metabolic thresholds, also critical power or velocity are applied to distinguish between heavy and severe intensity domains (Jamnick et al. 2020). Although the potential benefits of threshold assessments are widely understood, there is no universally accepted gold-standard method for defining the entire exercise intensity spectrum (Chavez-Guevara et al. 2024), which may lead to the application of more general fixed exercise intensities, e.g., those relative to maximum heart rate (HR).

Exercise intensity domains are defined according to physiological responses observed during incremental exercise testing. The first metabolic threshold is typically at the intensity corresponding to the first increase in blood lactate (i.e., LT1) or the point where the ventilation starts to increase in relation to VO_2_ (i.e., VT1) (Meyer et al. 2005; MacIntosh et al. 2021). In turn, the second metabolic threshold refers often to the maximal metabolic steady state. After this point, the blood lactate starts to accumulate more rapidly, and the ventilation accelerates also in relation to carbon dioxide production (Keir et al. 2024). Intensity domains have clearly distinct characteristics, and the domains differ substantially in terms of physiological responses, fatigue mechanisms (Burnley and Jones 2007; Iannetta et al. 2022), and maximum acute or chronic doses of exercises (Matomäki et al. 2024). Therefore, it would be important to define the domains as precisely as possible, so that the physiological strain could be accurately targeted and made comparable between individuals.

While the application of threshold-based intensity domains is suggested to be a superior method in standardizing exercise in relation to metabolic stress (Egger et al. 2016; Mann et al. 2013; Scharhag-Rosenberger et al. 2010), also the chronic training effects seem to be more precisely targeted with this type of approach (Meyler et al. 2021). There is evidence that the threshold-based individual exercise prescription would lead to superior training adaptations and decrease the likelihood of low training response (Weatherwax et al., 2019; Wolpern et al., 2015). It has even been suggested that fixed training intensities (e.g., based on maximum HR or VO_2_) could be among the major determinants affecting interindividual differences in training adaptations (Mann et al. 2014; Meyler et al. 2021). Although certain fixed percentages of the maximum could generally be correct, there are substantial interindividual differences at which percentage of the maximum the metabolic thresholds are located (Iannetta et al. 2020). This makes it challenging to find universal limits that would work across individuals of differing training status, age and sex.

Although the laboratory-defined intensity classifications can be regarded as the gold-standard method, it is not feasible to analyze metabolic thresholds in all contexts. For example, in the context of health-related physical activity, the intensity (e.g., light, moderate, and vigorous) is more commonly classified according to absolute metabolic equivalents (MET) or relative to the individual’s maximum heart rate (HR), HR reserve (HRR), or oxygen consumption (VO_2_) (Coates et al. 2023). In addition, the rating of perceived exertion (RPE) has been proposed as a feasible option for exercise prescription (Lehtonen et al. 2022). While there are general guidelines on how the exercise intensity should be classified according to these parameters (American College of Sports Medicine 2021), it is not clear where each of them stand in relation to the lactate thresholds. Earlier, Iannetta et al. (2021) have demonstrated with a large heterogeneous dataset how fixed MET values perform in defining intensity domains. To refine exercise prescription in cases where metabolic thresholds cannot be assessed, it could be beneficial to further examine how accurately each of fixed methods perform in the estimation of lactate thresholds, and which of them should be preferred if the aim is to standardize the physiological strain as accurately as possible.

To the best of our knowledge, no studies have examined and compared the accuracy of different fixed intensity anchors to estimate lactate thresholds. Therefore, the purpose of this study was first to examine how the lactate thresholds are positioned in relation to RPE, running speed, HR, HRR, VO₂, and VO_2_ reserve (VO₂_RES_) in recreational female and male runners. Second, the intent was to evaluate how accurately different fixed intensity anchors can estimate lactate thresholds in terms of external (i.e., running speed) and internal (i.e., HR) load, when sex-specific mean values of above parameters were applied.

## Methods

### Participants

The current data consist of pooled baseline test results from several studies that have been performed in the same laboratory. The participants were healthy females aged 20–50 years (*n* = 64) and males aged 20–45 years (*n* = 101), all of whom were accustomed to regular running. Using the McKay classification framework (McKay et al. 2022), the participants can be classified as trained/developmental (tier 2). The descriptive characteristics of the participants are presented in Table 1. All participants gave their written consent, and all study protocols were approved by the ethics committee of the University of Jyväskylä.

**Table 1:** Descriptive characteristics (mean ± SD) of the participants

Characteristic	Females *n* = 64	Males *n* = 101	All *n* = 165
Age (yrs)	36 ± 7	36 ± 6	36 ± 6
Body mass (kg)	62 ± 7	80 ± 10	73 ± 12
Height (cm)	165 ± 6	180 ± 5	174 ± 10
Fat (%)	23.3 ± 5.7	14.3 ± 4.3	17.8 ± 6.6
VO_2max_ (ml/kg/min)	44.1 ± 4.5	51.3 ± 5.9	48.5 ± 6.4
VO_2max_ classification (1–7)	6.1 ± 1.0	5.8 ± 1.1	5.9 ± 1.0
vPeak (km/h)	14.4 ± 1.2	16.9 ± 1.4	15.9 ± 1.8
HRmax (bpm)	187 ± 8	189 ± 8	188 ± 8
HRrest (bpm)	57 ± 6	50 ± 7	53 ± 7
Blood lactate max (mmol/l)	9.8 ± 2.9	11.0 ± 2.2	10.5 ± 2.5

VO_2max_ classification according to age-specific reference values by Shvartz and Reibold (1990). HRrest (resting heart rate) was defined as a 4-h nocturnal average. vPeak = maximum running speed during the incremental treadmill test; HRmax = maximum heart rate during the incremental treadmill test; Blood lactate max = maximum blood lactate during the incremental treadmill test.

## Study design

All participants performed an incremental treadmill test by running until volitional exhaustion. The test day was always preceded by at least one day of rest. The participants were advised to avoid heavy meals and caffeine 3–4 h before the test to avoid any gastrointestinal symptoms or other possible influences on measured variables. The body fat percentage was assessed with the skinfold method as a sum of four skinfolds (Durnin and Rahaman 1967) during the treadmill test visit (*n* = 86) or with the bioimpedance method during a separate visit (*n* = 79) at fasted state (InBody770-analyser, Biospace Co. Ltd., Seoul, Korea).

### Incremental treadmill test

The incremental treadmill test protocol used in this study has been the recommended testing protocol by the general Finnish fitness test guidelines (Keskinen et al. 2018), and it is widely applied in Finland for research and athletic testing. Although there are several protocols targeted especially for the determination of metabolic thresholds, the current protocol was considered as a viable option to assess the thresholds and maximum performance within the same test (Bentley et al. 2007). The test was conducted running on a treadmill (Telineyhtymä Oy, Kotka, Finland, *n* = 137; or H/P/Cosmos Saturn 300/100 r, *n* = 28). The test consisted of 3-min stages with a constant incline (0.5 degrees) and speed. The treadmill was stopped (~ 15–25 s) after each stage for the fingertip blood lactate measurement, after which the speed increased by 1 km/h. The starting speed was 7–10 km/h, which was expected to allow at least two stages before LT1. The VO_2_ was measured breath by breath (OxygonPro, Jaeger, Hoechberg, Germany, *n* = 62; Jaeger VyntusTM CPX, CareFusion Germany 234 GmbH, Hoechberg, Germany. *n* = 103) and averaged over the last 60 s of each stage. The HR was monitored with a Garmin HRM-Tri sensor that was paired with Garmin Forerunner 920XT watch (Garmin Ltd, Schaffhausen, Switzerland, *n* = 24) or Garmin Forerunner 245 M watch (*n* = 74), or with a Polar H10 sensor (Polar Electro, Kempele, Finland) that was paired with Polar Vantage V2 watch (*n* = 67). For each stage, the HR was averaged over the last 30 s. The perceived exertion was estimated after each stage with a Finnish translation of 6–20 RPE scale (Borg 1982). The VO_2max_ was defined as the highest 60-s mean oxygen consumption, and the maximum HR as the highest recorded (non-erroneous) value during the test (Tanaka et al. 2001). The maximum running speed (vPeak) of the test was defined as the speed at the last completed stage, or if the last stage was not finished, as the speed of the last completed stage (km/h) + (running time (s) of the unfinished stage – 30 s)/(180 – 30 s) × 1 km/h.

In addition to the measured maximum values (vPeak and HRmax), the same parameters were also derived using mathematical formulas. The HRmax was estimated with the formula of Tanaka et al. (2001) (208–0.7 × age). The vPeak was estimated with the formula of ACSM submaximal treadmill running test as described by Marsh (2012): A linear regression line between HR and running speed was drawn using the data points where the HR was greater than 110 bpm but below 85% of the age-predicted HRmax. Thereafter, the regression line was extrapolated to the speed corresponding to the age-predicted HRmax. Since 15 participants (4 males, 11 females) did not have at least two data points below 85% of the age-predicted maximum HR, data from altogether 150 participants were used in these analyses.

### Lactate threshold assessment

**Fig. 1:**
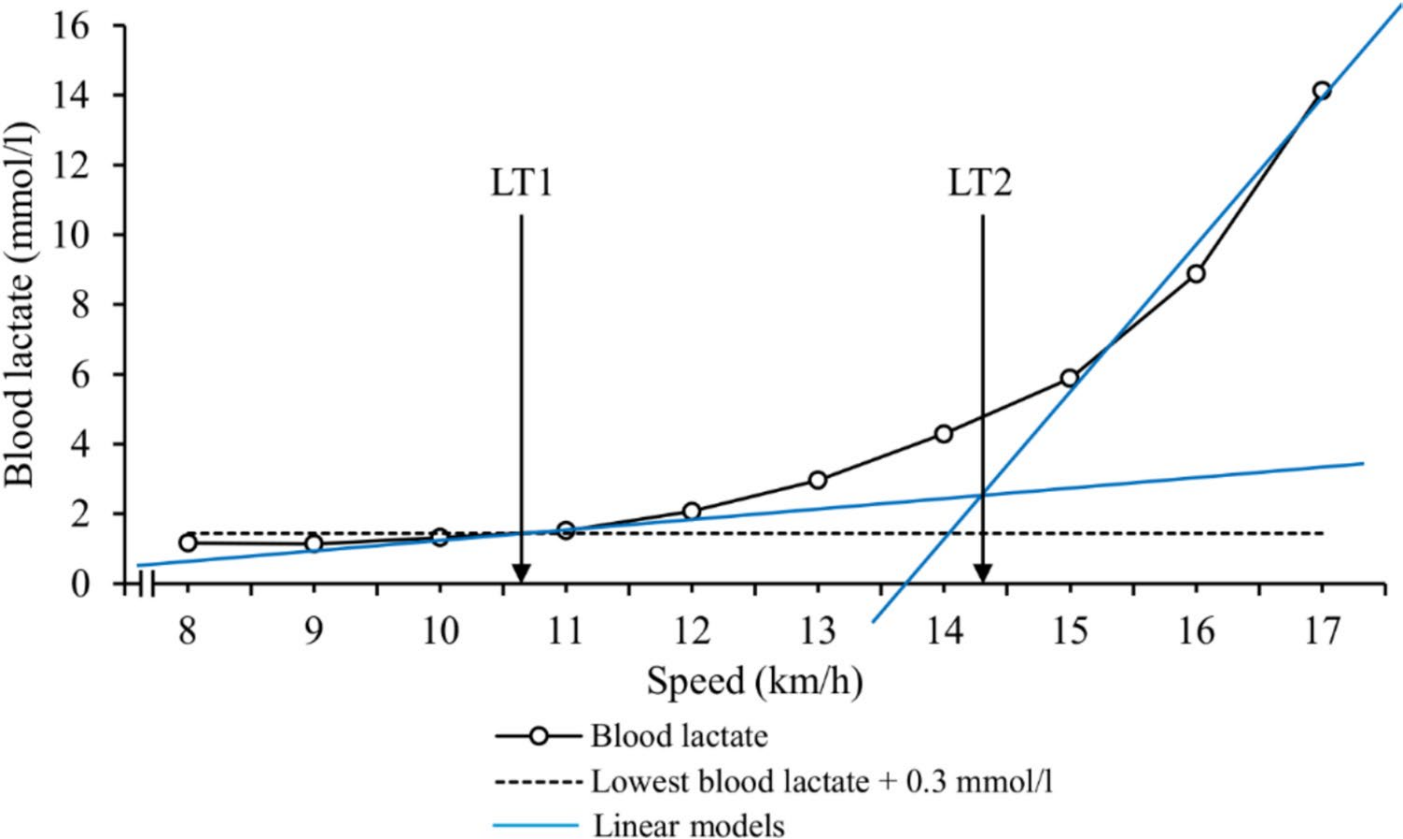
Determination of the first (LT1) and the second (LT2) lactate thresholds. LT1 was set at 0.3 mmol/l above the lowest lactate value and LT2 at the intersection point between (1) a linear model between LT1 and the next lactate value and (2) a linear model for the lactate points with the lactate increase of at least 0.8 mmol/l

Running speeds at the LT1 and LT2 were determined based on the change in the inclination of the blood lactate curve during the test (Nuuttila et al. 2021, 2024) according to general Finnish fitness test guidelines. The method and criteria for the thresholds are illustrated in Fig. 1.

Several parameters were analyzed at the lactate thresholds. In the case of the threshold positioning between the stages, the results were interpolated to estimate the value at the threshold. For the calculation of HRR and VO_2RES_, resting values were subtracted from the maximum values. A fixed value of 3.5 ml/kg/min was used as the basal VO_2_ (Jetté et al. 1990). The HRrest was analyzed from the nocturnal HR recordings (a 4-h period starting 30 min after going to sleep). The methodology of HR measurements varied slightly between the studies, but the results were derived either as an individual average of the control/preparatory period (*n* = 141) or during four separate nights preceding laboratory exercises (*n* = 24). The HR recordings were performed with an ECG-based Firstbeat Bodyguard 2 device (Firstbeat Technologies LTD, Jyväskylä, Finland, *n* = 98) and a photoplethysmography-based Polar Vantage V2 device (*n* = 67). The HR, VO_2_ and running speed were reported in relation to the individual maximum values (%) achieved during the test, while the HRR and VO_2RES_ were reported as a fraction (%) of the individual reserve.

## Statistical analysis

The results are presented as mean ± standard deviation (SD). The differences between males and females were compared with an unpaired samples t-test. The 95% confidence intervals for different parameters at LT1 and LT2 were calculated by multiplying the SD with 1.96. The estimated speed and HR at LT1 and LT2 were analyzed separately for each parameter using sex-specific mean values for each parameter. The Bland–Altman plot was used to analyze the 95% limits of agreement (LoA) for each estimation method. In addition, the mean absolute error (MAE) and mean absolute percentage errors (MAPE) were determined. Since the fitness status has been suggested to influence the location of ventilatory thresholds (Benítez-Muñoz et al. 2024a), and thereby also the error magnitude of the fixed anchors, the Pearson correlation coefficient was used to analyze the associations between the vPeak and relative intensities or the RPE at LT1 and LT2. These analyses were performed separately for females and males. All Statistical analyses were performed with Microsoft Excel 2016 (Microsoft Corporation, Redmond, WA, USA).

## Results

The mean values at the lactate thresholds are presented in Table 2. The relative values of running speed, HR, HRR, VO_2_ and VO_2RES_ at LT1 were higher in females compared to males (*p* < 0.001). In turn, regarding LT2, the females had higher relative HR and HRR, while the males had higher absolute blood lactate values (*p* < 0.001).

**Table 2:** Mean values (± SD) at the first (LT1) and second (LT2) lactate thresholds

	Females *n* = 64	Males *n* = 101	All *n* = 165
LT1			
Speed (%/vPeak)	67.3 ± 4.4	64.6 ± 4.6#	65.7 ± 4.7
HR (%/max)	83.6 ± 4.2	78.9 ± 4.4#	80.7 ± 4.9
HRR (%)	76.5 ± 6.1	71.3 ± 6.0#	73.3 ± 6.5
VO_2_ (%/max)	75.3 ± 5.6	70.8 ± 5.1#	72.5 ± 5.7
VO_2RES_ (%)	73.1 ± 6.1	68.7 ± 5.4#	70.4 ± 6.0
RPE (6–20)	12.8 ± 1.5	12.3 ± 1.6	12.5 ± 1.6
Bla (mmol/l)	1.6 ± 0.5	1.7 ± 0.4	1.7 ± 0.5
LT2			
Speed (%/vPeak)	80.9 ± 4.0	80.8 ± 3.4	80.8 ± 3.6
HR (%/max)	91.7 ± 2.2	89.9 ± 2.4#	90.6 ± 2.5
HRR (%)	88.1 ± 3.1	86.3 ± 3.4#	87.0 ± 3.4
VO_2_ (%/max)	87.8 ± 4.7	86.6 ± 3.6	87.1 ± 4.1
VO_2RES_ (%)	86.7 ± 5.0	85.6 ± 3.8	86.1 ± 4.4
RPE (6–20)	15.2 ± 2.2	15.5 ± 1.4	15.4 ± 1.8
Blood lactate (mmol/l)	2.9 ± 0.6	3.3 ± 0.6#	3.2 ± 0.6

*vPeak* Peak treadmill test speed, *HR* Heart rate, *HRR* Heart rate reserve, *VO*_*2*_ Oxygen consumption *VO*_*2RES*_ Oxygen consumption reserve, *RPE* Rating of perceived exertion

^#^p < 0.05 different from females

**Fig. 2:**
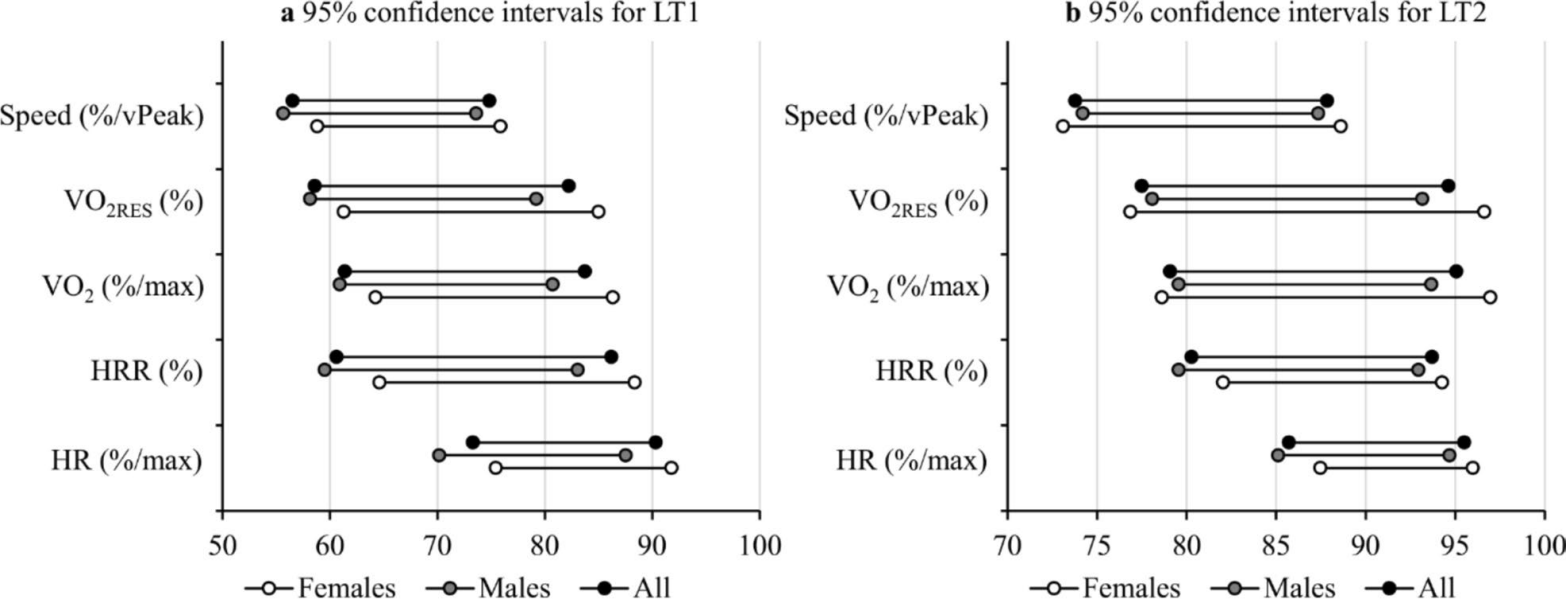
95% confidence intervals for **a** the first (LT1) and **b** the second lactate thresholds (LT2). HR = heart rate, HRR = heart rate reserve, VO_2_ = oxygen consumption VO_2RES_ = oxygen consumption reserve

**Fig. 3:**
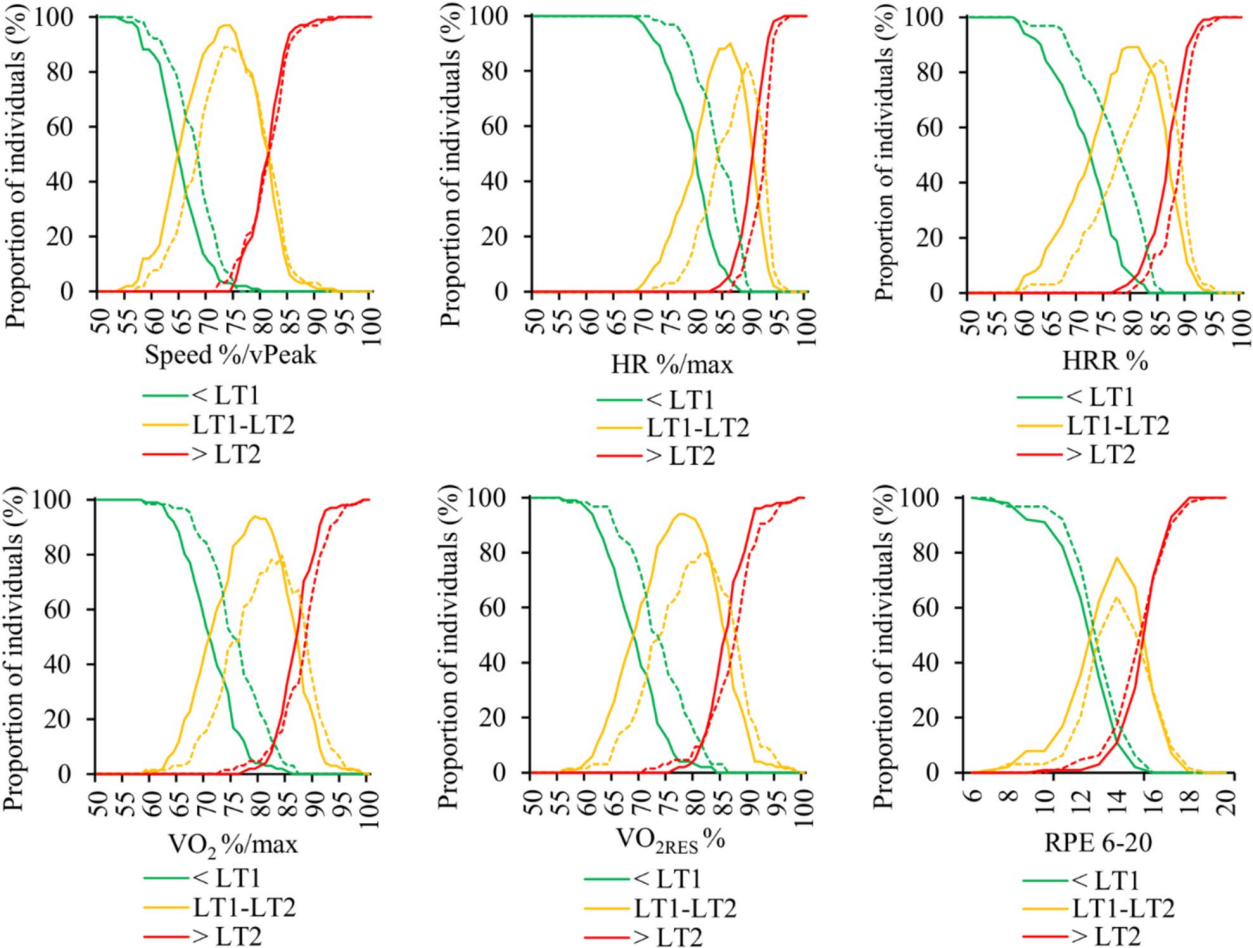
Distribution of individuals being below the first lactate threshold (LT1), between LT1 and the second lactate threshold (LT2) and above LT2 at given fixed results. Distributions are presented separately for females (dotted line) and males (solid line). vPeak = peak treadmill test speed, HR = heart rate, HRR = heart rate reserve, VO_2_ = oxygen consumption, VO_2RES_ = oxygen consumption reserve, RPE = rating of perceived exertion

**Fig. 4:**
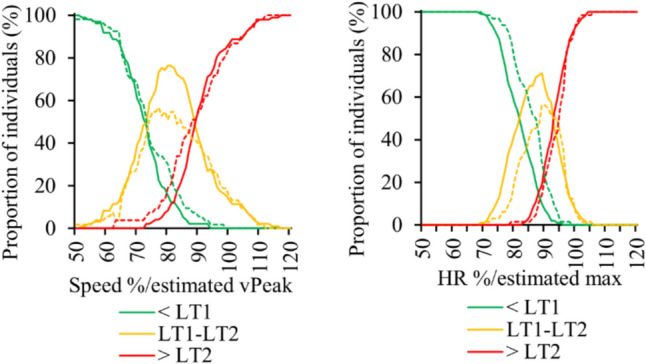
Distribution of individuals being below the first lactate threshold (LT1), between LT1 and the second lactate threshold (LT2) and above LT2 at given fixed results in relation to estimated maximum. Distributions are presented separately for females (dotted line) and males (solid line). vPeak = peak treadmill test speed, HR = heart rate

Figure 2 illustrates the 95% confidence intervals for the positioning of both thresholds. Regarding the RPE, the 95% confidence intervals at LT1 were from 9.4 to 15.7 and at LT2 from 11.0 to 19.8 in the total group of participants. Figure 3 demonstrates the distribution of different intensity domains at given fixed values. The corresponding distributions with estimated HRmax and vPeak are shown in Fig. 4.

The mean bias, LoA, MAE, and MAPE for the estimated thresholds are presented in Tables 3 and 4. In turn, the Bland–Altman plots for each analysis are provided as supplementary materials (ESM2-5). There were no significant differences between sexes with any of the methods (*p* > 0.05); thus, pooled results combining both sexes are presented. The running speed-derived estimation had the lowest error magnitude and LoAs for LT1 and LT2, while the RPE showed the highest error magnitude for both thresholds.

**Table 3:** Mean bias, the 95% limits of agreement (LoA), mean absolute error (MAE) and mean absolute percentage error (MAPE) of different averaging methods for the estimation of speed at the first (LT1) and second lactate thresholds (LT2) in pooled data of females and males

	Bias (km/h)	LoA (km/h)	MAE (km/h)	MAPE (%)
LT1				
Speed	0.0 ± 0.7	−1.4;1.4	0.6 ± 0.5	5.3 ± 4.2
RPE	−0.1 ± 1.1	−2.2;2.0	0.8 ± 0.7	8.3 ± 6.9
HR	−0.1 ± 0.9	−1.9;1.7	0.8 ± 0.5	7.5 ± 5.6
HRR	−0.1 ± 1.0	−2.0;1.8	0.8 ± 0.5	7.7 ± 5.6
VO_2_	0.0 ± 0.9	−1.7;1.8	0.7 ± 0.5	6.7 ± 5.0
VO_2RES_	0.0 ± 0.9	−1.7;1.7	0.7 ± 0.5	6.7 ± 5.0
LT2				
Speed	0.0 ± 0.6	−1.1;1.1	0.4 ± 0.4	3.4 ± 2.8
RPE	−0.1 ± 0.9	−1.9;1.7	0.8 ± 0.6	6.1 ± 4.6
HR	−0.1 ± 0.7	−1.3;1.2	0.5 ± 0.4	4.0 ± 3.1
HRR	−0.1 ± 0.7	−1.3;1.2	0.5 ± 0.4	4.0 ± 3.2
VO_2_	0.0 ± 0.7	−1.3;1.3	0.5 ± 0.4	3.9 ± 3.5
VO_2RES_	0.0 ± 0.7	−1.3;1.3	0.5 ± 0.4	3.9 ± 3.5

*HR* Heart rate, *HRR h*eart rate reserve, *VO*_*2*_ Oxygen consumption *VO*_*2RES*_ Oxygen consumption reserve, *RPE* Rating of perceived exertion

**Table 4:** Mean bias, the 95% limits of agreement (LoA), mean absolute error (MAE) and mean absolute percentage error (MAPE) of different averaging methods for the estimation of HR at the first (LT1) and second lactate thresholds (LT2) in pooled data of females and males

	Bias (bpm)	LoA (bpm)	MAE (bpm)	MAPE (%)
LT1				
Speed	−0.1 ± 6.6	−13.0;12.8	4.9 ± 4.4	3.3 ± 3.0
RPE	−1.2 ± 9.7	−20.1;17.7	7.4 ± 6.3	5.1 ± 4.5
HR	0.1 ± 8.1	−15.7;15.9	6.6 ± 4.6	4.3 ± 3.0
HRR	0.0 ± 8.2	−15.9;15.9	6.7 ± 4.6	4.4 ± 3.0
VO_2_	0.1 ± 8.0	−15.5;15.8	6.1 ± 5.1	4.1 ± 3.3
VO_2RES_	−0.1 ± 8.0	−15.7;15.5	6.1 ± 5.1	4.1 ± 3.4
LT2				
Speed	−0.2 ± 3.7	−7.5;7.5	2.8 ± 2.5	1.6 ± 1.5
RPE	−1.2 ± 6.5	−13.9;11.5	5.2 ± 4.1	3.1 ± 2.6
HR	0.0 ± 4.6	−8.6;8.7	3.4 ± 2.8	2.0 ± 1.6
HRR	0.0 ± 4.6	−8.8;8.7	3.4 ± 2.8	2.0 ± 1.7
VO_2_	0.0 ± 4.4	−8.6;8.6	3.3 ± 2.9	1.9 ± 1.7
VO_2RES_	-0.1 ± 4.4	−8.7;8.5	3.3 ± 2.9	1.9 ± 1.7

*HR* Heart rate, *HRR* Heart rate reserve, *VO*_*2*_ Oxygen consumption *VO*_*2RES*_ Oxygen consumption reserve, *RPE* Rating of perceived exertion

The effects of using estimated vPeak and HRmax values on the mean bias, LoA, MAE and MAPE are presented in the supplementary materials (ESM1). In comparison to any of the measured maximal values or the RPE (Table 3 and Table 4), the accuracy of fixed thresholds was poorer when estimated maximal values were used (to determine the threshold intensities).

Table 5 presents the associations between the vPeak and relative intensities or the RPE at LT1 and LT2. The only significant correlations were found for LT1 and VO_2_%/max (*r* = −0.284, *p* = 0.023) and VO_2res_% (*r* = −0.258, *p* = 0.040) in females and for LT1 and VO_2_%/max (*r* = −0.227, *p* = 0.022) in males.

**Table 5:** Pearson correlation coefficients between vPeak (km/h) and relative intensity or RPE at the first (LT1) and second (LT2) lactate thresholds

	LT1	LT2
Females		
Speed (%/vPeak)	−0.06	−0.13
RPE (6–20)	0.15	−0.05
HR (%/max)	−0.08	0.12
HRR (%)	−0.02	0.17
VO_2_ (%/max)	−0.28*	−0.18
VO_2RES_ (%)	−0.26*	−0.16
Males		
Speed (%/vPeak)	0.08	0.16
RPE (6–20)	0.13	0.10
HR (%/max)	−0.09	−0.06
HRR (%)	0.00	0.03
VO_2_ (%/max)	−0.23*	−0.08
VO_2RES_ (%)	−0.19	−0.06

*HR* Heart rate, *HRR* Heart rate reserve, *VO*_*2*_ Oxygen consumption *VO*_*2RES*_ Oxygen consumption reserve, *RPE* Rating of perceived exertion

*statistical significance *p* < 0.05

## Discussion

The current study investigated the positioning of lactate thresholds in recreational female and male runners and evaluated how accurately different fixed intensity anchors could estimate lactate thresholds in terms of external (running speed) and internal (heart rate) load. The main findings were that LT1 occurred at a higher relative intensity (HR, speed and VO_2_) in females compared to males, suggesting that fixed intensity anchors should be considered separately for females and males. Furthermore, the running speed-derived lactate threshold estimations provided the most accurate results, while the RPE-derived estimations were the least accurate. As expected, the estimation of maximum values impaired the prediction accuracy significantly, showing that the optimal method also depends on the availability of the maximum values.

In the context of endurance training, exercise intensity is typically controlled using HR, speed, the power of exercise, or RPE level (Bellinger et al. 2020). Acute and chronic responses to endurance training are often studied using fixed values relative to the maximum (80%/HRmax). According to research evidence, however, the individually determined training intensity based on the metabolic thresholds could help standardize the metabolic stress of training between individuals (Meyler et al. 2021) and induce greater training adaptations (Meyler et al. 2024). The present study highlighted the potential challenges of using fixed intensity, if the aim of training is to induce comparable physiological strain between individuals. For instance, at an intensity of 80%/HRmax that is quite often applied in exercise interventions, 57% of the participants were exercising at the moderate intensity domain while 43% at the heavy intensity domain. Therefore, when using fixed anchors, it is important to choose methods and intensities that allow the most standardized stimulus between individuals. It is also critical to acknowledge the difference between intensities fixed according to estimated vs. measured values, since the use of estimated maximum values increases the magnitude of error significantly. Furthermore, when considering issues such as the heterogeneity of training responses or dose–response models, the potential challenges arising from the methods of defining the dose should be addressed.

In the current study, the approximate lactate thresholds as percentages of maximum HR and speed were at 80% and 65% at LT1, and at 90% and 80% at LT2, respectively. In previous studies, relative intensities at the lactate thresholds have varied. For example, Iannetta et al. (2020) reported lower percentages, LT1 at 75%/HRmax and 60%/VO_2max_ in healthy adults, and Weltman et al. (1990) higher percentages, LT1 at 88%/HRmax and 83%/VO_2max_ in trained runners. In turn, a recent study of Benítez-Muñoz et al. (2024a, b) reported very similar HR, VO_2_, and speed relative to maximum at VT1 and VT2 compared to the LT results of this study. An interesting finding in the present study was that LT1 appeared to be at a higher relative speed, HR, and VO_2_ along with LT2 at higher relative HR in females compared to males. The results were slightly in contrast to those reported by Gaskill et al. (2023) who found no sex differences in VT1 when the fitness level was standardized, which itself might affect lactate thresholds (Weltman et al. 1989, 1990). On the other hand, Iannetta et al. (2020), Støa et al. (2020), and Benítez-Muñoz et al. (2024a, b) reported similarly differing lactate threshold values between sexes, and that is why, the discrepancies might also relate to different analysis methods (i.e., ventilatory vs. lactate threshold). While the current data cannot explain the exact reasons behind the sex differences, the HR-based differences could hypothetically be associated with the lower cardiac output of females, largely due to their generally lower blood volume and oxygen carrying capacity (Diaz-Canestro et al. 2022). Furthermore, Benítez-Muñoz et al. (2024a, b) speculated that the sex difference of the thresholds might relate to greater reliance on fat oxidation at low-to-moderate intensities as well as greater proportion of the type 1 muscle fibers in females. Sex differences have also been reported in deoxygenation during fatiguing exercise, which in turn could contribute to variations in exercise tolerance and intensity-duration relationship (Ansdell et al. 2019). In any case, it seems that the use of fixed HR prescription (e.g., 80% or 90%/HRmax) is likely to lead males exercising at a higher intensity than females. Therefore, it should be more critically considered, whether similar exercise intensity recommendations apply to both sexes. If ignored, the observed sex differences may even have practical consequences for the training adaptations in the form of lesser or suboptimal stimuli in females undertaking exercise at the same fixed intensity or similar prescription as males (Ansdell et al. 2020).

The confidence intervals of the different lactate threshold estimation methods were quite wide (Fig. 2), demonstrating that the average-based values alone may not be very effective at the individual level. Interestingly, the lower limit of the confidence intervals for LT1 appeared to align closely with the moderate-to-vigorous transitions proposed by the ACSM in terms of HR, HRR, VO_2_ and VO_2RES_ (American College of Sports Medicine 2021). In practice, these results suggest that if the aim is to ensure moderate intensity exercise prescription for a recreational runner, the intensity should be ≤ 55%/vPeak, ≤ 70%/HRmax, or ≤ 60%/VO_2max_. On the other hand, based on the upper limit of the confidence intervals for LT2, the relative intensity would need to be quite high (e.g., ≥ 90%/vPeak or ≥ 95%/HRmax) to ensure that the participants are exercising in the severe intensity domain. Overall, standardizing training at the heavy intensity domain seems challenging with fixed methods, as there was no fixed intensity that would have allowed all individuals to be exercising within the same domain. To maximize the proportion of individuals at the heavy intensity domain, the current data suggest the approximate values of 75%/vPeak, 80%/VO_2max_, and 85%/HRmax. It may be useful to note that critical speed can also be used to define the maximal metabolic steady state and the heavy-severe intensity boundary (Jones et al. 2019). Since critical speed can be estimated even with a single-visit field test (Galbraith et al. 2014) without the need for lactate or ventilatory measurements in a laboratory setting, it can be a feasible option for defining intensity domains more accurately for recreational runners. As a limitation in assessing the entire intensity spectrum, the moderate-heavy intensity boundary (i.e., LT1) cannot be accurately estimated from the critical speed only (Hunter et al. 2024).

When the lactate threshold estimations were based on the sex-specific mean values, the MAPE for the running speed ranged from 5.3% (speed) to 8.3% (RPE) at LT1, and from 3.4% (speed) to 6.1% (RPE) at LT2. Based on these results, it seems that the location of LT2 can be more accurately estimated than the location of LT1. Especially, the estimates of LT2 appear surprisingly accurate, considering the daily variability and reliability of metabolic threshold assessments (Pallarés et al. 2016). The results also suggest that fixing the exercise intensity according to the external speed or power output relative to the maximum is the most suitable method if a direct measurement of thresholds is not possible. However, it should be underlined that the error substantially increased when the maximum speed was estimated using the ACSM’s submaximal treadmill test formula. Fixing the thresholds as percentages of the estimated vPeak increased the MAPE to 9.2% at LT1 and 8.2% at LT2. If the assessment of the maximum performance and metabolic thresholds is not possible, the RPE-based prescription may perform better than HR- or speed-derived methods, as highlighted by the smaller MAPE in RPE-based threshold determination when compared to estimated maximal speed or HRmax-derived thresholds. Although the RPE did not perform particularly well as an independent method, it could potentially aid in determining the intensity if the HR range is also described with the corresponding target RPE (Lehtonen et al. 2022). The precision of RPE in exercise prescription may also depend on the individual’s training experience (Johnson et al. 2017), and therefore, less experienced individuals may require more thorough induction into the RPE. In addition to the actual RPE, at least athletes may have the ability to assess the maximal metabolic steady state in self-paced trials by estimating the highest sustainable intensity they can maintain for the intended duration (e.g., 30 min) (Mattioni Maturana et al. 2017).

Given the importance of determining the exercise intensity domains and the challenges with fixed thresholds demonstrated in the present study, it would be beneficial to find feasible ways to estimate intensities with sufficient accuracy. Regarding the performance estimations, studies have focused more on VO_2max_, while indirect threshold estimation has received somewhat less attention. Indirect and submaximal methods are mainly related to subjective methods (Bok et al. 2022) such as the “talk test” (Reed and Pipe 2014) or “Rabbit-test” (Giovanelli et al. 2020). Another option is to use non-invasive physiological markers such as HR- (Jones and Doust 1997) or HR variability-based thresholds (Kaufmann et al. 2023). Indirect methods usually provide reasonably accurate estimates and at least moderate correlations with the actual thresholds, but they do not seem to perform significantly better than the fixed intensity anchors reported in the present study. Thus, the LoA, MAE, and MAPE results of this study can also be considered in the future as reference level for assessing the accuracy of the estimates provided by such indirect methods. If a new method is not more accurate than the average-based vPeak or HR estimates, it is probably not worth using. As wearable technologies advance, new options for indirect threshold estimation (even live-monitoring of thresholds) are likely to emerge (Andriolo et al. 2024). Moreover, the training data recorded with wearables could help determine critical speed (Smyth et al. 2022) without laboratory testing.

The current study involved a large, pooled dataset from five separate studies performed in the same laboratory. It is important to emphasize that the participants were recreationally trained, and the threshold values from this study should not be applied directly to different populations. Interestingly, Gaskill et al. (2023) reported that VT1 occurred at a higher relative intensity in well-trained and sedentary individuals, while they were lower in moderately trained individuals, indicating a U-shaped pattern between the fitness level and threshold positioning. On the other hand, Benítez-Muñoz et al. (2024a, b) found that the VO_2max_ did not correlate with the %/VO_2max_, %/HRmax or %/vPeak at ventilatory thresholds. However, the higher the training status (classified as low, medium, high), the higher the relative fraction of the maximum was. Similar to Benítez-Muñoz et al. (2024a, b), no strong correlations were found between fitness levels (vPeak) and the relative location of the thresholds in the current study. Nevertheless, it is likely there would be more differences if the participants’ exercise backgrounds were more heterogeneous than in this study. Since these studies have been cross-sectional by nature, it is not possible to argue that the thresholds would shift due to training adaptations, and consequently, longitudinal studies are needed to examine the adaptability of such characteristics. When interpreting the results of this study, it is also relevant to consider the potential impact of the testing mode (e.g., bicycle ergometer vs. running (Vainshelboim et al. 2020)) and the testing protocol (Bentley et al. 2007) on the thresholds. Therefore, the current absolute or relative results may not necessarily apply universally to different testing modes or protocols. Finally, the thresholds were analyzed as lactate thresholds, and it is acknowledged that different threshold determination methods have their strengths and weaknesses (Faude et al. 2009; Jamnick et al. 2020). It is important to be aware of the daily variability associated with gold-standard methods, which can easily be overlooked when evaluating alternative methods. In the future, large database studies could help to understand in a more detailed manner the effects of testing protocols and various background factors on the location of intensity domains. This would allow the creation of more tailored exercise intensity guidelines that can also take the target group into account.

## Conclusions

In conclusion, the current study found that LT1 is positioned at a higher relative intensity in females than males, suggesting that same fixed values should not be used for both sexes. The mean percentage errors for fixed thresholds varied between 3.4% and 8.3%, and the speed-based methods seemed to provide the most accurate estimations for lactate thresholds when the maximum values were measured. The estimation of maximum values impaired the prediction accuracy significantly, and the RPE-based method performed better than those based on the estimated maximum HR or vPeak. Therefore, the optimal intensity prescription method also depends on the availability of the maximum values. Further studies should address similar methods in different target populations and with different testing modes to critically evaluate how general exercise intensity guidelines should be formed.

## Supplementary Information

Below is the link to the electronic supplementary material.

Supplementary file1 (DOCX 21 KB)

Supplementary file2 (DOCX 589 KB)

Supplementary file3 (DOCX 615 KB)

Supplementary file4 (DOCX 614 KB)

Supplementary file5 (DOCX 596 KB)

## Data Availability

The datasets generated and/or analyzed during the current study are available from the corresponding author on reasonable request.

## Abbreviations

HIT: High-intensity training
HR: Heart rate
HRR: Heart rate reserve
LIT: Low-intensity training
LT1: The first lactate threshold
LT2: The second lactate threshold
MAE: Mean absolute error
MAPE: Mean absolute percentage error
MIT: Moderate-intensity training
MET: Metabolic equivalent
RPE: Rating of perceived exertion
VO2: Oxygen uptake
VO2max: Maximum oxygen uptake
VO_2RES_: Oxygen uptake reserve
vPeak: Maximum treadmill test speed
VT1: The first ventilatory threshold
VT2: The second ventilatory threshold
