# Supplementary material for: The accuracy of fixed intensity anchors to estimate lactate thresholds in recreational runners
Source: Eur J Appl Physiol. 2025 Mar 15;125(8):2161–71. doi: 10.1007/s00421-025-05748-8 (PMC12354492; doi:10.1007/s00421-025-05748-8)
Supplement: Supplementary file 1 — Supplementary file1 (DOCX 21 KB) [file 421_2025_5748_MOESM1_ESM.docx]

The accuracy of fixed intensity anchors to estimate lactate thresholds in recreational runners

European Journal of Applied Physiology

Olli-Pekka Nuuttila^1,2^, Piia Kaikkonen^3^, Harri Sievänen^1^, Tommi Vasankari^1,4^, Heikki Kyröläinen^2^

1 The UKK Institute for Health Promotion Research, Kaupinpuistonkatu 1, FI-33500 Tampere Finland

2 Faculty of Sport and Health Sciences, University of Jyväskylä, Jyväskylä, Finland

3 Tampere Research Center of Sports Medicine, UKK Institute, Kaupinpuistonkatu 1, 33500 Tampere, Finland

4 Faculty of Medicine and Health Technology, Tampere University, Tampere, Finland

**Corresponding author:**

Olli-Pekka Nuuttila

Email: [olli-pekka.nuuttila@ukkinstituutti.fi](mailto:olli-pekka.nuuttila@ukkinstituutti.fi)

| Electronic supplementary material 1. Mean bias, the 95% limits of agreement (LoA), mean absolute error (MAE) and mean absolute percentage error (MAPE) for the estimation of HR and speed at the first (LT1) and second lactate thresholds (LT2). The results are derived from estimated HRmax and vPeak. | | | | |
| --- | --- | --- | --- | --- |
|  | Bias | LoA | MAE | MAPE (%) |
| **LT1 speed (km/h)** |  |  |  |  |
| Estimated vPeak | 0.1 ± 1.3 | -2.4;2.6 | 1.0 ± 0.8 | 9.2 ± 7.5 |
| Estimated HRmax | 0.1 ± 1.3 | -2.4;2.5 | 1.0 ± 0.7 | 9.9 ± 7.4 |
| **LT2 speed (km/h)** |  |  |  |  |
| Estimated vPeak | 0.1 ± 1.4 | -2.6;2.8 | 1.1 ± 0.9 | 8.2 ± 6.7 |
| Estimated HRmax | 0.0 ± 1.2 | -2.4;2.4 | 1.0 ± 0.7 | 7.9 ± 5.6 |
| **LT1 HR (bpm)** |  |  |  |  |
| Estimated vPeak | -0.1 ± 10.6 | -20.8;20.6 | 8.4 ± 6.4 | 5.6 ± 4.2 |
| Estimated HRmax | 0.5 ± 10.0 |  | 8.7 ± 5.7 | 5.8 ± 3.8 |
| **LT2 HR (bpm)** |  |  |  |  |
| Estimated vPeak | 0.0 ± 8.6 | -16.8;16.9 | 6.7 ± 5.3 | 4.0 ± 3.1 |
| Estimated HRmax | -0.3 ± 8.0 | -16.0;15.5 | 6.6 ± 4.6 | 3.9 ± 2.7 |
| LT1 = The first lactate threshold, LT2 = The second lactate threshold, vPeak = peak treadmill test speed, HR = heart rate, MAE = mean absolute error, MAPE = mean absolute percentage error. | | | | |
