# Supplementary material for: The accuracy of fixed intensity anchors to estimate lactate thresholds in recreational runners
Source: Eur J Appl Physiol. 2025 Mar 15;125(8):2161–71. doi: 10.1007/s00421-025-05748-8 (PMC12354492; doi:10.1007/s00421-025-05748-8)
Supplement: Supplementary file 4 — Supplementary file4 (DOCX 614 KB) [file 421_2025_5748_MOESM4_ESM.docx]

The accuracy of fixed intensity anchors to estimate lactate thresholds in recreational runners

European Journal of Applied Physiology

Olli-Pekka Nuuttila^1,2^, Piia Kaikkonen^3^, Harri Sievänen^1^, Tommi Vasankari^1,4^, Heikki Kyröläinen^2^

1 The UKK Institute for Health Promotion Research, Kaupinpuistonkatu 1, FI-33500 Tampere Finland

2 Faculty of Sport and Health Sciences, University of Jyväskylä, Jyväskylä, Finland

3 Tampere Research Center of Sports Medicine, UKK Institute, Kaupinpuistonkatu 1, 33500 Tampere, Finland

4 Faculty of Medicine and Health Technology, Tampere University, Tampere, Finland

**Corresponding author:**

Olli-Pekka Nuuttila

Email: [olli-pekka.nuuttila@ukkinstituutti.fi](mailto:olli-pekka.nuuttila@ukkinstituutti.fi)


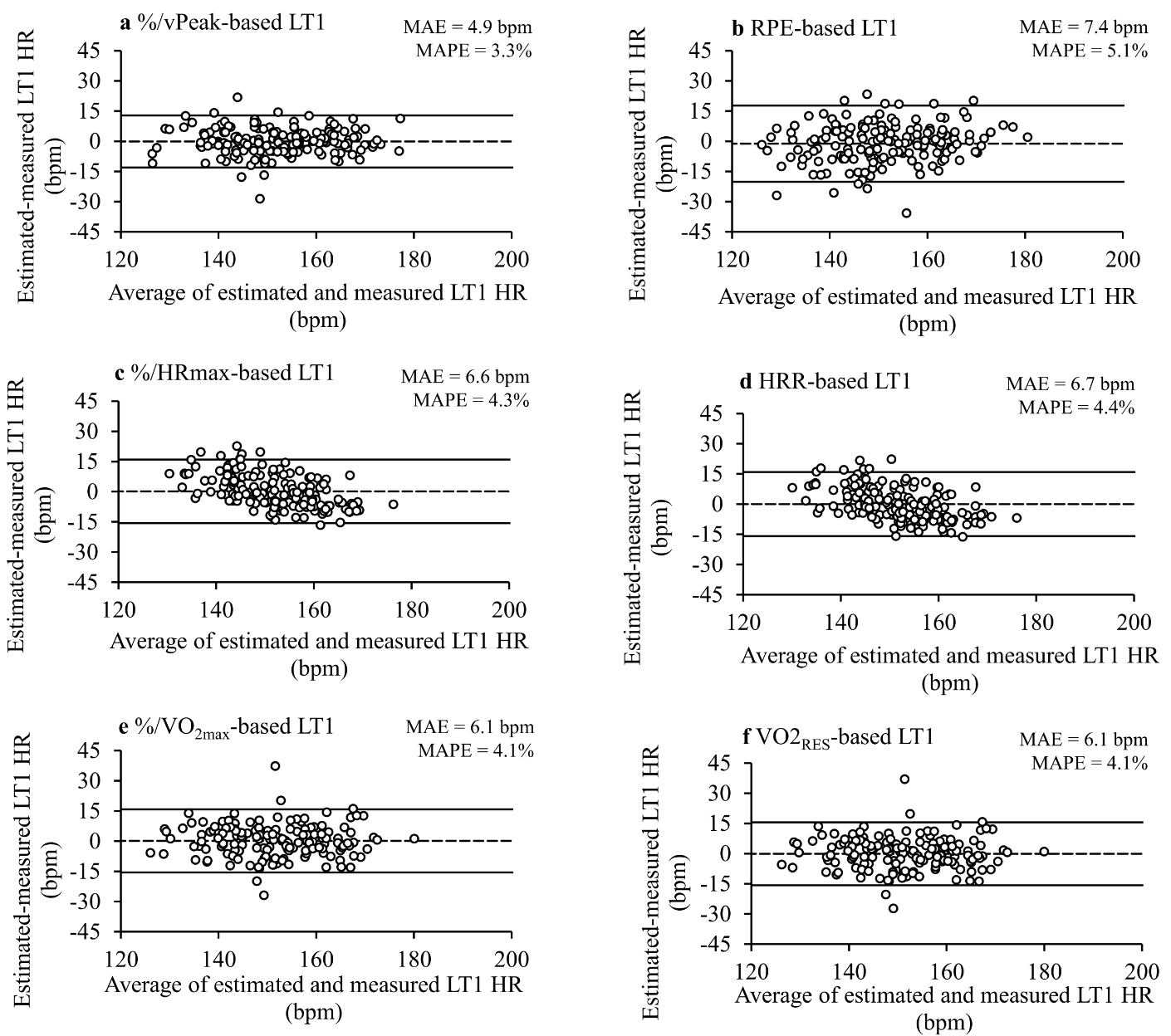


Electronic supplementary material 4. Bland-Altman plots for estimated heart rate (HR) at the first lactate threshold (LT1). vPeak = peak treadmill test speed, HRR = heart rate reserve, VO_2_ = oxygen consumption VO_2RES_ = oxygen consumption reserve, RPE = rating of perceived exertion, MAE = mean absolute error, MAPE = mean absolute percentage error.
